# Prostaglandin receptor EP4 expression by Th17 cells is associated with high disease activity in ankylosing spondylitis

**DOI:** 10.1186/s13075-019-1948-1

**Published:** 2019-06-28

**Authors:** Charlotte Klasen, Anja Meyer, Paula S. Wittekind, Iris Waqué, Schafiq Nabhani, David M. Kofler

**Affiliations:** Division of Clinical Immunology and Rheumatology, Department I of Internal Medicine, University of Cologne, Kerpenerstr. 62, 50937 Cologne, Germany

**Keywords:** Ankylosing spondylitis, Th17 cells, *PTGER4*, Interleukin-23 receptor, FoxO1, Rheumatoid arthritis, Prostaglandin receptor EP4

## Abstract

**Background:**

Th17 cells are involved in the pathogenesis of ankylosing spondylitis (AS). However, the mechanism underlying enhanced Th17 cell accumulation in AS remains unknown. The prostaglandin E_2_ receptor EP2/EP4 signaling pathway plays a critical role in the development of autoimmune Th17 cells. Interestingly, recent genome-wide association studies (GWAS) have identified five risk alleles for AS in *PTGER4*, the gene encoding for EP4. The aim of this study was to reveal a possible link between EP4 and disease activity in patients with AS.

**Methods:**

Th17 cells from patients with AS were analyzed for the transcriptional expression of prostaglandin receptor genes by quantitative RT-PCR. Th17 cells from patients with rheumatoid arthritis (RA) and from healthy individuals served as controls. EP4 receptor expression in Th17 cells was assessed ex vivo by flow cytometry and by western blot. Functional analysis using EP4-specific agonists was performed to reveal how EP4 regulates Th17 cells.

**Results:**

EP4 is significantly overexpressed in Th17 cells from patients with AS compared to Th17 cells from healthy individuals or patients with RA or psoriatic arthritis (PsA). EP4 upregulation is unique to Th17 cells and is not found in other CD4^+^ T cell subsets. Specific activation of EP4 drives Th17 cell development and promotes EP4 expression in a positive feedback loop in AS but not in RA or PsA. Mechanistically, EP4 acts via upregulation of the interleukin-23 receptor (IL-23R), by suppressing the RORγt inhibitor FoxO1 and by enhancing STAT3 phosphorylation. Increased EP4 expression levels in Th17 cells from AS patients correlate with high disease activity as defined by a Bath Ankylosing Spondylitis Disease Activity Index (BASDAI) score ≥ 4 (*r* = 0.7591, *p* = 0.0016).

**Conclusions:**

EP4 is a potential marker of disease activity in patients with AS. Aberrant EP4 expression might contribute to pathogenic Th17 cell accumulation and represent a new target for the treatment of AS.

**Electronic supplementary material:**

The online version of this article (10.1186/s13075-019-1948-1) contains supplementary material, which is available to authorized users.

## Background

Ankylosing spondylitis (AS) is a multifactorial autoimmune disease leading to new bone formation and ankylosis of affected joints. The susceptibility to develop AS is influenced by genetic factors. The strongest association with AS has been found for the *HLA-B27* allele [1]. Recent genome-wide association studies (GWAS) have identified the single nucleotide polymorphisms (SNPs) rs10440635, rs9283753, rs16869602, rs12186979, and rs13354346 in the prostaglandin receptor EP4 gene *PTGER4* as risk alleles for AS [2–4]. Moreover, another risk allele for AS has been found in a gene involved in prostaglandin synthesis, providing strong evidence for a pathogenic role of prostaglandin E_2_ (PGE_2_) and its receptor EP4 in AS [5]. So far, the functional basis for this genetic association remains unknown.

Th17 cells are believed to be involved in the pathogenesis of AS and anti-IL-17A-specific antibodies have been recently established as an effective treatment [6–8]. Other therapies targeting Th17 cells in AS, including Janus kinase inhibitors, are currently under investigation [9, 10]. The frequency of Th17 cells and the serum concentrations of the Th17-related cytokines interleukin-17 (IL-17) and interleukin-23 (IL-23) are significantly elevated in the peripheral blood of AS patients [11–15]. Interestingly, the amount of Th17 cells is higher in male patients compared to female patients and increases during anti-tumor necrosis factor α (TNFα) therapy, probably due to redistribution of Th17 cells from inflamed joints [16, 17]. In contrast to patients with AS, the percentage of circulating Th17 cells is significantly reduced in patients with early non-radiographic axial spondyloarthritis and negatively correlates with disease activity [18].

The implication of Th17 cells in AS pathogenesis is further supported by the observation that Th17 cells show a disease-specific miRNA signature, including the expression of the Th17 cell regulator miR-10b-5p [19]. Furthermore, it has been reported that the genetic variants K528R and Q730E in *ERAP1* determine the strength of Th17 cell responses in AS [20]. It remains unknown which mechanisms trigger Th17 cell accumulation in AS. PGE_2_ can induce Th17 cells through EP2 and EP4 receptor signaling [21–26]. It has been recently shown that EP2 and EP4 signaling is critical in Th17-mediated autoimmune inflammation of the skin [25]. So far, the specific role of PGE_2_ in AS remains unknown. Since several genetic variants in the EP4 gene are associated with AS susceptibility, the aim of this study was to explore a possible link between EP4 and disease activity in patients with AS.

## Methods

### Study subjects

All patients with AS fulfilled the modified New York AS criteria [27]. Patients with RA fulfilled the 2010 ACR/EULAR classification criteria [28]. All patients were untreated for at least 8 weeks prior to inclusion in the study. Blood samples from age- and sex-matched healthy individuals were used as controls. Blood was drawn after written informed consent was obtained in accordance with the Declaration of Helsinki following a study protocol approved by the Ethics Committee of the University of Cologne [29].

### Human T cell isolation

Primary human lymphocytes were isolated from peripheral blood from patients with AS, RA, PsA, and SLE and healthy controls by Pancoll® density gradient centrifugation (PAN™-Biotech GmbH, Aidenbach, Germany). CD4^+^ T cells were purified by negative selection using the CD4^+^ T cell isolation kit. Where indicated, CD45RA^+^RO^−^ naïve CD4^+^ T cells were magnetically sorted using CD45RA MicroBeads and the autoMACS pro device (all Miltenyi Biotec, Bergisch Gladbach, Germany). The purity of the sorted cell populations was verified by flow cytometry and was at least 96%. Viable cells were counted using the Vi-CELL XR cell counter (Beckman Coulter, Krefeld, Germany) or the automated cell counter CellCountess (Life Technologies GmbH, Darmstadt, Germany).

### Th17 polarization

Where indicated, CD45RA^+^RO^−^ naïve CD4^+^ T cells were cultured in X-Vivo 15 (Lonza, Cologne, Germany) media supplemented with 1% human serum and 1% penicillin-streptomycin (both Sigma-Aldrich, Saint Louis, USA). Cells were cultured for 4 days with the T cell Activation/Expansion Kit (Miltenyi Biotec) and recombinant human TGF-β (5 ng/μl; PAN™-Biotech GmbH, Aidenbach, Germany), IL-1β (12.5 ng/μl), IL-6 (25 ng/μl; both Miltenyi Biotec), and IL-23 (25 ng/μl; PeproTech, Rocky Hill, USA). Medium and cytokines were refreshed after 3 days.

### T cell subset induction

Where indicated, CD4^+^ T cells were cultured in X-Vivo 15 (Lonza, Cologne, Germany) media supplemented with 1% human serum and 1% penicillin-streptomycin (both Sigma-Aldrich, Saint Louis, USA) Cells were cultured for 4 days with the T cell Activation/Expansion Kit (Miltenyi Biotec). For Th0 cell induction recombinant human IL-2 (20 U; Miltenyi Biotech) was added. For Th1 cell induction, recombinant human IL-12 (10 ng/ml; Miltenyi Biotec) and anti-human IL-4 mAb (10 μg/ml; BioLegend) were added. For Th2 cell induction, recombinant human IL-4 (10 ng/ml; PeproTech, Rocky Hill, USA) and anti-human IFNγ mAb (10 μg/ml; BioLegend) were added. For Th9 cell induction, recombinant human IL-4 (10 ng/ml; PeproTech, Rocky Hill, USA), recombinant human TGF-β (5 ng/μl; PAN™-Biotech GmbH, Aidenbach, Germany), and anti-human IFNγ mAb (10 μg/ml; BioLegend) were added. For iT_reg_ cell induction, recombinant human TGF-β (5 ng/μl; PAN™-Biotech GmbH, Aidenbach, Germany) and recombinant human IL-2 (20 U; Miltenyi Biotech) were added as described previously [30].

### Prostaglandin E2 receptor (EP2/EP4) stimulation

CD4^+^ T cells were stimulated for 3 days with the EP4 agonist misoprostol, the EP2 agonist butaprost or with prostaglandin E_2_ at a concentration of 1 μM each.

### Flow cytometry

CD4^+^ T cells were stimulated with PMA (100 ng/ml) and ionomycin (1.5 μM; both Cell Signaling Technology®, Danvers, USA) in the presence of Brefeldin A (eBioscience, San Diego, USA) for 3 h. To exclude dead cells, cells were stained by the LIVE/DEAD™ Fixable Dead Cell Stain Kit (Invitrogen, ThermoFisher Scientific, Carlsbad, USA). For intracellular staining, cells were fixed and made permeable by the BD Cytofix/Cytoperm Kit (BD Bioscience, Heidelberg, Germany) according to the manufacturer’s instructions and stained with anti-IL17A, anti-IFN-γ (both affymetrix eBioscience), anti-IL4, anti-IL9, anti-CD127, anti-CD25 (all from BioLegend Inc., San Diego, USA), anti-EP2, anti-EP4 receptor (both Columbia Bioscience, Frederick, USA), and anti-IL23R (ThermoFisher Scientific, Carlsbad, USA). Th17 cells were identified by IL-17A expression in purified CD4^+^CD45RO^+^RA^−^ T cells. For detection of phosphorylated proteins, cells were fixed using the BD PhosFlow Fix Buffer I and made permeable by the BD PhosFlow Permbuffer III (both BD Bioscience, Heidelberg, Germany) according to the manufacturer’s instructions. The cells were then stained with anti-STAT3 and anti-pSTAT3 (pY705) antibodies. For staining of FoxO1, cells were fixed by 4% formaldehyde (Carl Roth GmbH, Karlsruhe, Germany) and made permeable by 90% methanol (PanReac AppliChem GmbH, Darmstadt, Germany). Cells were then stained with anti-FoxO1 antibodies. Data were acquired on the Gallios 10/3 flow cytometer (Beckman Coulter, Krefeld, Germany).

### Western blot analysis

CD4^+^ cells from AS, RA, PsA, and SLE patients and healthy controls were incubated under Th17 skewing conditions (see the “Th17 polarization” section) with misoprostol, butaprost, or prostaglandin E_2_ at a concentration of 1 μM each for 4 days. Cells were lysed with the cell lysis buffer, and protein concentration was established with the BCA Protein Assay Kit (both Cell Signaling Technology®, Danvers, USA). Lysates were run on 4–15% polyacrylamide gels (Bio-Rad Laboratories, Munich, Germany). Blotting was performed with the Trans-Blot® Turbo™ Transfer System (Bio-Rad Laboratories). Proteins were detected with the following antibodies: β2-microglobulin (D8P1H) rabbit mAb, GAPDH (D16H11) rabbit mAb (both Cell Signaling Technology®), EP4 Receptor (c-Term) rabbit pAb (Cayman Chemical, Ann Arbor, USA), and anti-rabbit IgG HRP-linked (Cell Signaling Technology®). Detection was performed using the INTAS ChemoStar software.

### Quantitative real-time PCR

RNA was isolated from CD45RA^+^RO^−^ T cells using the RNeasy Mini Kit and converted into cDNA using the QuantiTectReverse Transcription Kit (both Qiagen, Hilden, Germany). All primers were purchased from Applied Biosystems. All reactions were performed using the 7500 Fast Real-Time PCR System (Applied Biosystems). The values are represented as the difference in C_t_ values normalized to β2-microglobulin for each sample using the following formula: relative RNA expression = (2-^dCt^) ×  10^3^.

### Enzyme-linked immunosorbent assay (ELISA)

ELISA for IL-23, IL-21, IL-6, IL-1β, and TGFβ1 were performed according to the manufacturer’s instructions (Invitrogen, ThermoFisher Scientific, Carlsbad, USA).

### Statistics

Statistical analysis was performed using GraphPad Prism. Where indicated, data were analyzed by non-parametric Mann-Whitney, Wilcoxon, Friedman, or Kruskal-Wallis test and are presented as the mean ± SEM. *p* < 0.05 was considered as statistically significant. Correlation was analyzed using Spearman rank.

## Results

**Fig. 1:**
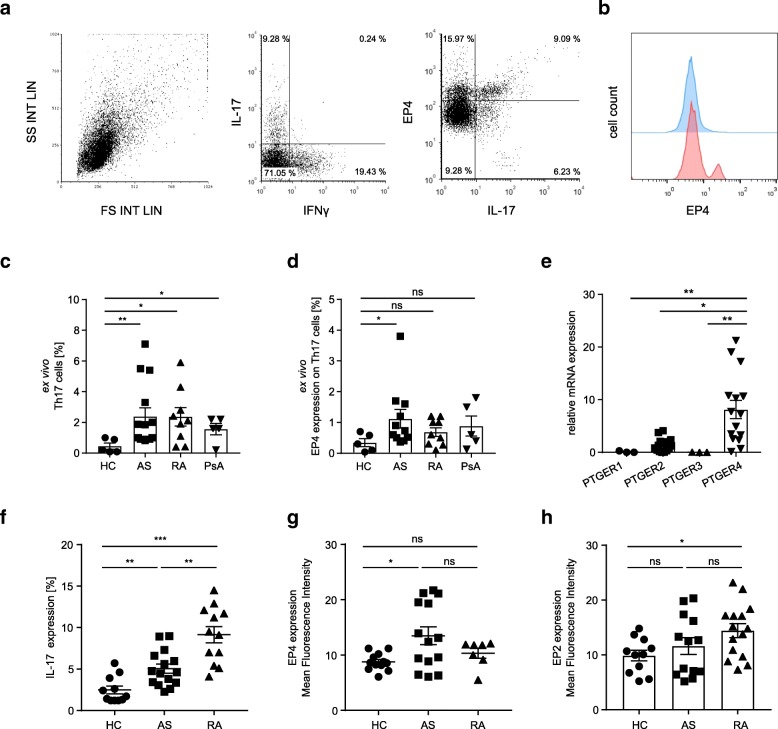
EP4 is overexpressed in Th17 cells from patients with ankylosing spondylitis. **a**, **b** Representative examples of flow cytometry analysis of EP4 expression. CD4^+^ T cells from the peripheral blood of patients with AS were purified using MACS technique, cultured under Th17-skewing conditions for 4 days and analyzed for the expression of IL-17, IFNγ, and EP4 (histogram: negative control = blue, EP4 expression = red). **c** Ex vivo flow cytometry analysis of Th17 cell frequencies and **d** ex vivo EP4 expression in Th17 cells from healthy controls (HC) and from patients with AS, RA, or PsA (HC *n* = 5, AS *n* = 14, RA *n* = 9, PsA *n* = 5; *p < 0.05, **p < 0.01; *p* value calculated using Mann-Whitney test). **e** RT-PCR analysis of PGE_2_ receptor genes in Th17 cells from patients with AS. Th17 cells were generated from naïve CD4^+^CD45RA^+^ T cells under Th17 skewing conditions (*n* = 15; **p < 0.01; *p* value calculated using Kruskal-Wallis test). The values are represented as the difference in C_t_ values normalized to β2-microglobulin for each sample using the following formula: relative RNA expression = (2-^dCt^) × 10^3^. **f** Percentage of IL-17-positive CD4^+^ T cells after 4 days of in vitro cell culture under Th17-skewing conditions (HC *n* = 11; AS *n* = 15; RA *n* = 12; **p < 0.01,***p < 0.001; *p* value calculated using Mann-Whitney test). **g** EP4 expression in Th17 cells (HC *n* = 12, AS *n* = 14, RA *n* = 7; *p < 0.05; *p* value calculated using Mann-Whitney test) and **h** EP2 expression in Th17 cells (HC *n* = 11, AS *n* = 13, RA *n* = 15 *p < 0.05, *p* value calculated using Mann-Whitney test) was assessed by flow cytometry in CD4^+^ T cells cultured under Th17-skewing conditions for 4 days. The percentage and the mean fluorescence intensity (MFI) of cells are expressed as mean ± SEM

Mononuclear cells were isolated from the peripheral blood of patients with AS and CD4^+^ T cells were purified using MACS technique. EP4 expression and cytokine expression in CD4^+^ T cells were then analyzed ex vivo by flow cytometry. In addition, CD4^+^ T cells were cultured in vitro for 4 days under Th17-skewing conditions. The patient characteristics are summarized in Table 1. In accordance with previous reports, ex vivo flow cytometry analysis revealed that the frequency of Th17 cells is increased in the peripheral blood of patients with AS compared to healthy controls (HC) (*p* = 0.0066, 2.34 ± 0.56% in AS vs. 0.4 ± 0.2% in HC) (Fig. 1a–c; Additional file 1: Figure S1a). In addition, the level of *IFNG* is increased in CD4^+^ T cells from AS patients (*p* = 0.0339) (Additional file 1: Figure S1b). Similar to patients with AS, patients with rheumatoid arthritis (RA) or psoriatic arthritis (PsA) have increased Th17 cell frequencies in the peripheral blood (Fig. 1c). Ex vivo analysis of EP4 in Th17 cells from patients with AS revealed a significantly higher level of EP4 expression as compared to healthy controls (*p* = 0.0380, 1.12 ± 0.3% in AS vs. 0.34 ± 0.13% in HC) (Fig. 1d). Interestingly, EP4 expression is not increased ex vivo in Th17 cells from patients with RA or PsA (Fig. 1d). RA and PsA are both rheumatic autoimmune diseases without any genetic evidence for an association of *PTGER4* with disease susceptibility. To assess prostaglandin E_2_ (PGE_2_) receptor expression on mRNA level, CD45RA^+^RO^−^ naïve CD4^+^ T cells were cultured for 4 days under Th17 skewing conditions. Expression levels of the PGE_2_ receptor genes *PTGER1*, *PTGER2*, *PTGER3,* and *PTGER4* were analyzed by RT-PCR. Compared to *PTGER4*, the expression of *PTGER2* is significantly lower in Th17 cells from patients with AS (Fig. 1e). *PTGER1* and *PTGER3* are not expressed. The level of *PTGER2* is not elevated in Th17 cells from patients with AS compared to healthy individuals (Additional file 1: Figure S1c). Flow cytometry analysis of in vitro cultured CD4^+^ T cells confirmed the increase in Th17 cell frequencies in patients with AS or RA (5.03 ± 0.5% in AS vs. 2.4 ± 0.47% in HC, *p* = 0.0027; 9.1 ± 0.98% in RA; Fig. 1f) and the EP4 receptor overexpression in AS (MFI 13.5 ± 1.5 in AS vs. 8.7 ± 0.45 in HC, *p* = 0.0139; Fig. 1g). Importantly, the expression of EP4 per cell (mean fluorescence intensity, MFI) and the percentage of EP4-positive cells are both significantly elevated in AS (Fig. 1g and Additional file 1: Figure S1d). In contrast, EP2 is elevated in Th17 cells from patients with RA but is not significantly increased in patients with AS (*p* = 0.0129; 14.4 ± 1.3 in RA vs. 9.9 ± 0.97 in HC; 11.6 ± 1.5 in AS; Fig. 1h). In RA, EP2 expression is increased compared to healthy controls after activation with PGE_2_ or with an EP4 agonist (*p* = 0.0402; Additional file 1: Figure S1e). In vitro and ex vivo analysis of other CD4^+^ T cell subsets revealed that significant EP4 expression is also found on protein and on mRNA level in Th0, Th1, Th2, Th9, and nTreg or iTreg cells (Additional file 2: Figure S2a-e). However, in these CD4^+^ T cell subsets, there is no difference in EP4 expression between AS patients and healthy controls. Th17 cells from AS patients are therefore the only CD4^+^ T cell subset with distinct EP4 expression compared to healthy individuals. Furthermore, EP4 is not significantly elevated in these CD4^+^ T cell subsets in patients with RA or PsA compared to healthy controls (Additional file 2: Figure S2a-d).

**Table 1:** Patient characteristics

Patient number	Sex	Age	Disease duration (years)	BASDAI	HLA-B27^+^
1	f	37	5	7.5	Positive
2	f	45	11	6.4	n.d.
3	f	25	3	7.3	Positive
4	f	73	25	5.7	Positive
5	m	35	2	8.5	Positive
6	f	57	24	6.1	n.d.
7	f	50	9	7.4	Positive
8	m	32	8	7.2	Positive
9	f	28	7	6.3	Positive
10	m	46	12	9.2	n.d.
11	m	23	4	8.2	Positive
12	f	26	5	2.6	Positive
13	m	37	2	1	Negative
14	f	63	3	6.2	Positive
15	m	51	14	n.d.	n.d.
16	m	52	16	5.3	n.d.
17	m	54	14	1.4	n.d.
18	f	43	9	5	n.d.
19	m	53	3	0.2	Positive
20	m	24	2	0.2	Positive
Mean ± SEM	n/a	42.7 ± 3.15	8.9 ± 1.55	5.35 ± 0.66	n/a

Blood samples were obtained from patients with ankylosing spondylitis (AS). Patients were either newly diagnosed with AS or untreated for at least 8 weeks. *n.d.* not determined, *n/a* not applicable

**Fig. 2:**
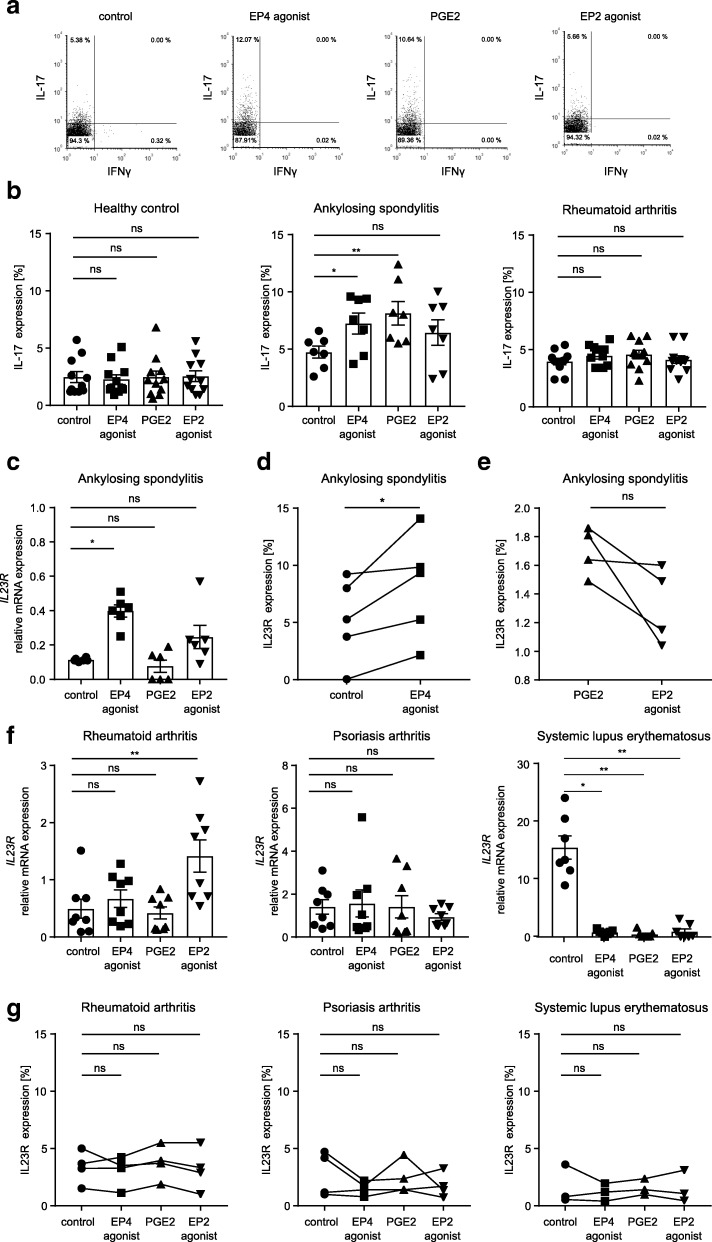
Specific activation of EP4 promotes Th17 cell expansion and upregulation of IL23 receptor in ankylosing spondylitis. **a** Representative example of flow cytometric analysis and **b** summary of the results of IL-17 measurement in purified CD4^+^ T cells from healthy controls (HC), patients with AS, and patients with RA after 4 days of in vitro cell culture in the presence of the EP4 agonist misoprostol, PGE_2_, or the EP2 agonist butaprost (HC *n* = 11; AS *n* = 8; RA *n* = 11; *p < 0.05, **p = < 0.01, *p* value calculated using Friedman test). **c** RT-PCR analysis of IL23R mRNA expression in CD4^+^ T cells from patients with AS after 3 days of in vitro stimulation with PGE2 or an EP4 or EP2 agonist. Data are shown as relative expression. **d** Flow cytometry analysis of IL-23 receptor expression in IL-17^+^ CD4^+^ T cells from patients with AS after 3 days of in vitro stimulation with an EP4 agonist (*p < 0.05; *p* value calculated using Wilcoxon test) or **e** after 3 days of stimulation with PGE_2_ or an EP2 agonist. **f** Analysis of IL23R expression by RT-PCR and by flow cytometry (**g**) in CD4^+^ T cells from patients with RA, PsA, and SLE after 3 days of in vitro stimulation with PGE_2_ or an EP4 or EP2 agonist. Data are shown as relative expression (RT-PCR: *n* = 6; *p < 0.05, **p < 0.01; flow cytometry: RA *n* = 5, PsA *n* = 4, SLE *n* = 3; *p* value was calculated using Friedman test). The percentage of positive cells is expressed as mean ± SEM

Our next aim was to reveal the role of EP4 in Th17 cell induction in AS. Therefore, we activated Th17 cells through the EP4 receptor by using the specific agonist misoprostol or by adding PGE_2_. We found that EP4-specific activation significantly drives the differentiation of Th17 cells from patients with AS while the percentage of Th17 cells from healthy controls or patients with RA is not increased by EP4-specific activation (4.9 ± 0.5% without stimulation vs. 7.0 ± 0.8% with an EP4 agonist and 7.8 ± 0.9% with PGE_2_ in AS; *p* = 0.0498 and *p* = 0.0201, respectively; Fig. 2a, b).

**Fig. 3:**
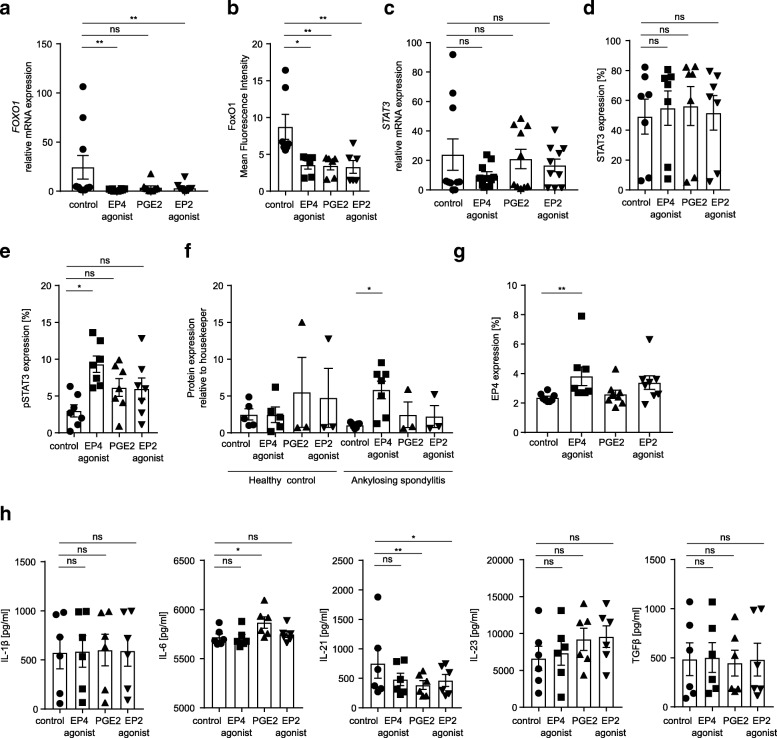
EP4 inhibits FoxO1, enhances Stat3 phosphorylation, and is upregulated in a positive feedback loop. **a** RT-PCR and **b** flow cytometric analysis of FoxO1 in CD4^+^ T cells from patients with AS after 3 days of in vitro stimulation with PGE2 or an EP4 or EP2 agonist (*n* = 10 for RT-PCR, *n* = 6 for flow cytometric analysis¸ *p < 0.05, **p < 0.01; *p* value calculated using Friedman test). **c** RT-PCR analysis of *STAT3* in CD4^+^ T cells from patients with AS after 3 days of in vitro stimulation with PGE_2_ or an EP4 or EP2 agonist (*n* = 10). **d** Flow cytometric analysis of STAT3 and **e** pSTAT3 expression in IL17^+^CD4^+^ T cells from patients with AS after 3 days of in vitro stimulation with PGE_2_ or an EP4 or EP2 agonist (*n* = 7, *p < 0.05; *p* value calculated using Friedman test). **f** EP4 upregulation in IL17^+^CD4^+^ T cells from patients with AS and from HC after 3 days of in vitro stimulation with PGE_2_ or an EP4 or EP2 agonist. Protein quantification of western blot analysis is shown and was performed using the LabImage software (*n* ≥ 3, *p < 0.05 calculated using Wilcoxon test). **g** Flow cytometry analysis of EP4 expression in IL17^+^CD4^+^ T cells from patients with AS. **h** Cell culture supernatants from CD4^+^ T cells from patients with AS were collected after 3 days of stimulation with PGE_2_ or an EP4 or EP2 agonist and analyzed by ELISA for the secretion of cytokines (*n* = 6, *p < 0.05, **p < 0.01, *p* value calculated using Friedman test). Data are shown as mean ± SEM

To further investigate the mechanisms underlying Th17 cell expansion through EP4 activation, we assessed the expression of interleukin-23 receptor (IL-23R). IL-23R signaling is known to induce pathogenic Th17 cells in autoimmune diseases, while non-pathogenic Th17 cells are induced by TGF-β [31]. We found that EP4 activation significantly upregulates IL-23R on mRNA level (0.1 ± 0.004 in the control group vs. 0.4 ± 0.03 after stimulation with an EP4 agonist; Fig. 2c). In addition, IL-23R is upregulated on protein level by an EP4-specific agonist (5.3 ± 1.6% in the control group vs. 8.1 ± 2.0% after stimulation with an EP4-specific agonist; Fig. 2d and Additional file 3: Figure S3a). In contrast to EP4, specific activation of EP2 induces no upregulation of IL-23R (Fig. 2e). In PsA, prostaglandin receptor stimulation has no influence on IL-23R expression on mRNA level or protein level (Fig. 2f, g). In RA, *IL23R* is upregulated on mRNA level but not on protein level by the EP2-specific agonist butaprost, while *IL23R* mRNA is decreased in systemic lupus erythematosus (SLE) by activation of EP2 or EP4 (Fig. 2f, g). In the next step, we assessed FoxO1 expression, as IL-23R expression is regulated by FoxO1, an inhibitor of RORγt [32]. We found a significant decrease in FoxO1 expression on mRNA level and on protein level after incubation of Th17 cells with an EP4 agonist, indicating a possible mechanism of action of EP4 receptor signaling (*p* = 0.0084, 24.4 ± 11.96 in the control group vs. 1.25 ± 0.32 after stimulation with an EP4 agonist and *p* = 0.0457, 8.7 ± 1.7 in the control group vs. 3.6 ± 0.5 after stimulation with an EP4 agonist, respectively; Fig. 3a, b). In contrast, STAT3, an important regulator of RORγt, is not significantly influenced by EP4 activation (Fig. 3c, d). However, phosphorylated STAT3 (pSTAT3) is significantly increased upon EP4-specific stimulation with the EP4 agonist misoprostol but not with an EP2-specific agonist or by PGE_2_ (*p* = 0.0029, 2.9 ± 0.82 in the control group vs. 9.31 ± 1.10 after stimulation with an EP4 agonist; Fig. 3e). Importantly, EP4-specific activation increases EP4 expression in a positive feedback loop in Th17 cells from patients with AS (*p* = 0.0058, 2.4 ± 0.1 in the control group vs. 3.8 ± 0.6 after EP4 stimulation and *p* = 0.0313, 1.05 ± 0.13 in the control group vs. 5.83 ± 1.20 after EP4 stimulation, respectively; Fig. 3f, g and Additional file 3: Figure S3b). This feedback loop is not observed in other rheumatic autoimmune diseases (RA, PsA, SLE) (Additional file 3: Figure S3c). To verify if EP4 stimulation has an influence on Th17-related cytokines, we analyzed IL-1β, IL-6, IL-21, IL-23, and TGFβ concentrations by ELISA in the supernatant of EP4-activated CD4^+^ T cells from patients with AS and found that EP4 activation has no influence on cytokine concentrations (Fig. 3h). Furthermore, there are no significant differences in cytokine expression on mRNA level after EP4-specific activation, while PGE_2_ enhances IL-1β and IL-23 mRNA expression (Additional file 4: Figure S4a). To exclude the possibility that EP4 upregulation in Th17 cells is induced by cell activation, we incubated Th17 cells from healthy individuals with various concentrations of anti-CD3 antibodies, with a combination of anti-CD2, anti-CD3, and anti-CD28 antibodies and with PMA/ionomycin. These experiments revealed that activation of T cells has no influence on EP4 expression (Additional file 4: Figure S4b).

**Fig. 4:**
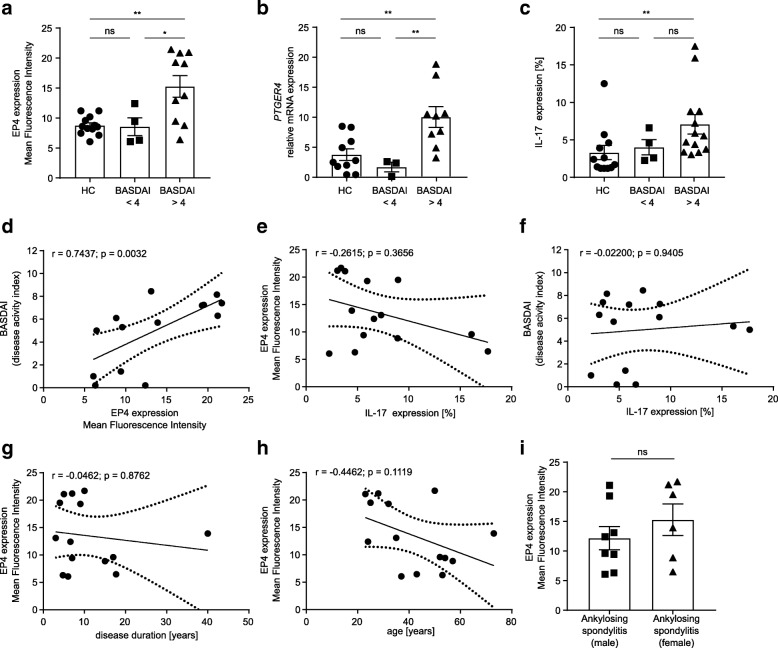
EP4 expression in Th17 cells is associated with high disease activity in AS. **a** EP4 expression levels in AS patients with low or high BASDAI values. EP4 expression was analyzed by flow cytometry in IL-17^+^ purified CD4^+^ T cells after 4 days of in vitro cell culture and is presented as MFI. **b** *PTGER4* expression in patients with low or high BASDAI values. *PTGER4* expression was assessed by RT-PCR. Th17 cells were induced in vitro for 4 days from naïve CD4^+^CD45RA^+^ T cells under Th17-skewing conditions. **c** IL-17 expression in AS patients with low or high BASDAI values. Cells were analyzed by flow cytometry. The percentage of positive cells is shown. **d** Correlation between BASDAI and EP4 expression in Th17 cells, **e** between EP4 and IL-17 expression, **f** between BASDAI and IL-17 expression as well as correlation of EP4 with **g** disease duration, **h** age of patients and **i** sex (same cells as in Fig. 1f were used). Spearman *r* correlation and Mann-Whitney test were used to determine the significance

In order to verify if EP4 expression correlates with disease activity, we assessed the Bath Ankylosing Spondylitis Disease Activity Index (BASDAI) score of each patient. A BASDAI score ≥ 4 is defined as high disease activity. We found that Th17 cells from patients with high disease activity express higher amounts of EP4, as shown by increased mean fluorescence intensity as well as by the percentage of EP4-positive cells (Fig. 4a and Additional file 4: Figure S4c). In contrast, EP4 expression in Th17 cells from patients with low disease activity does not differ from healthy controls (MFI 15.5 ± 1.8 in patients with BASDAI ≥ 4 vs. 8.5 ± 1.5 in patients with BASDAI < 4, *p* = 0.036 and 8.8 ± 0.5 in healthy controls; *p* = 0.005, respectively; Fig. 4a and Additional file 4: Figure S4c). A significant difference in EP4 expression between healthy controls and patients with high disease activity was also observed on mRNA level (*p* = 0.0041; Fig. 4b). While patients with high disease activity have significantly higher numbers of circulating Th17 cells in the peripheral blood compared to healthy controls, the frequency of Th17 cells is not useful to discriminate between patients with low disease activity and patients with high disease activity (*p* = 0.0276; Fig. 4c). However, the level of EP4 expression in Th17 cells seems to be very useful to distinguish between both groups of patients as the level of EP4 strongly correlates with disease activity measured by BASDAI score (*r* = 0.7437 *p* = 0.0032; MFI is shown in Fig. 4d, the percentage of positive cells is shown in Additional file 4: Figure S4d, e). Importantly, a strong correlation between BASDAI and EP4 is found in both, in vitro and ex vivo analyzed Th17 cells (Additional file 4: Figure S4d, e). In contrast, the frequency of Th17 cells in the peripheral blood does not correlate with EP4 expression (*r* = − 0.2615, *p* = 0.3656; Fig. 4e) or with the BASDAI score (*r* = 0.02200, *p* = 0.9405; Fig. 4f). Furthermore, there is no correlation between EP4 expression and disease duration or age of the patients (Fig. 4g, h) and no association with sex (Fig. 4i).

## Discussion

The implementation of disease activity assessment scores has revolutionized the evaluation of treatment response in AS. In 1994, the Bath Ankylosing Spondylitis Functional Index (BASFI) and the BASDAI were developed at the Royal National Hospital for Rheumatic Diseases in Bath, UK [33, 34]. To obtain a better instrument for the assessment of disease activity, the Assessment of SpondyloArthritis international Society (ASAS) developed a new score containing laboratory parameters [35]. The Ankylosing Spondylitis Disease Activity Score (ASDAS) includes the C reactive protein (CRP) or the erythrocyte sedimentation rate (ESR) or both to discriminate between high and low disease activity [35, 36]. Elevated CRP or ESR is present in about 40–50% of patients with AS [37]. Importantly, CRP and ESR can be elevated under various conditions and are not specific for AS. Therefore, there is an unmet need for additional biomarkers that reflect disease activity in AS. Laboratory parameters that have been associated with disease severity include IL-6, IL-17, and the matrix metalloproteinase (MMP) profile [12, 38, 39]. Here we report that EP4 expression levels in Th17 cells can be used to discriminate between patients with high and low disease activity. Importantly, elevated levels of EP4 are found in patients with AS but not in patients with RA. Therefore, EP4 expression in Th17 cells might be a more specific biomarker for disease activity in AS and could help to improve assessment of treatment response by laboratory parameters, e.g., in clinical trials.

Our findings reveal that EP4 is selectively increased in Th17 cells from patients with AS, thereby providing a possible explanation for the association between *PTGER4* gene variants and AS susceptibility. Although the odds ratio for each of the *PTGER4* risk alleles is low, the accumulation of five risk alleles within one gene region points towards an implication of EP4 in the pathogenesis of AS. The odds ratios of the risk alleles might add to an increased overall association between EP4 and AS. In addition, the association might be further enhanced by epigenetic factors, as shown by epigenetic fine-mapping of autoimmune disease-associated enhancer RNA (eRNA) located near the *PTGER4* gene [40]. *PTGER4* might also contribute to the pathogenesis of AS by increasing osteoblastic activity and bone formation [41–43]. Importantly, we did not observe significantly increased EP4 expression levels in Th17 cells from patients with RA, a rheumatic disease without an association with *PTGER4* gene variants. The level of ex vivo EP4 expression is similar or even significantly higher in other CD4^+^ T cell subsets from patients with AS. However, Th17 cells are the only CD4^+^ T cell subsets with a higher expression of EP4 compared to healthy controls. This points towards a possible pathogenic role of EP4 in Th17 cells, while it seems to have physiological roles in other CD4^+^ T cell subsets.

**Fig. 5:**
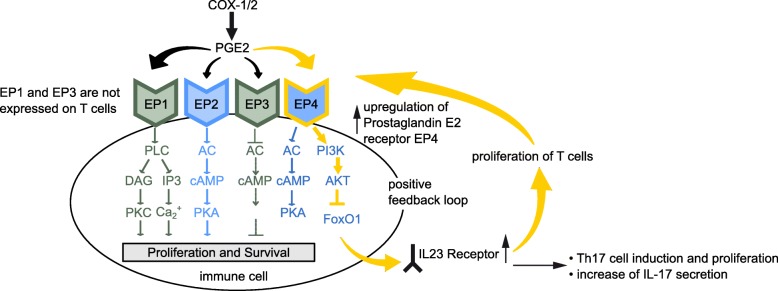
Model of EP4 involvement in the generation of pathogenic Th17 cells in AS. PGE_2_ binds to the EP4 receptor and inhibits FoxO1 expression in Th17 cells. As a result, the IL-23 receptor (IL-23R) is upregulated. Activation of IL-23 receptor leads to Th17 cell accumulation and to an upregulation of EP4 in a positive feedback loop. The figure was adapted from Rundhaug et al. [45]

We show that PGE_2_-induced Th17 cell development is mainly mediated through EP4 and not through EP2 in AS. This unique role of EP4 can be explained by different signaling pathways of EP2 and EP4. While the receptors share the cAMP pathway, EP4 signaling additionally activates PI3K/AKT, a pathway that controls Th17 cell differentiation [44–48] (Fig. 5). It has been shown that PI3K/AKT inhibits FoxO1 [49]. Remarkably, as we have observed, FoxO1 expression is reduced after EP4-specific activation (Fig. 3a). As they activate different signaling pathways, EP2 and EP4 receptors have at least in part distinct functions in immune inflammation. EP4 signaling promotes IL-23 production by dendritic cells, while EP2 activation suppresses IL-23 production [50]. Moreover, EP2 and EP4 differentially regulate IL-17A and IL-17F secretion by Th17 cells [51]. Interestingly, PGE_2_ levels are increased in the peripheral blood of patients with high disease activity but not in the blood of patients with low disease activity [52]. Compared to AS, EP4 plays a less significant role in Th17 cell induction in RA (Additional file 2: Figure S2b). The findings are consistent with clinical observations showing a less important role for Th17 cells and a smaller therapeutic effect of anti-IL-17A targeting therapies in RA, resulting in FDA approval of anti-IL-17A antibodies for the treatment of AS but not for the treatment of RA [7]. The significant lower amount of EP4 in several CD4^+^ T cell subsets from patients with RA and PsA suggests that EP4-mediated Th17 cell expansion could be a disease-specific mechanism in the pathogenesis of AS.

Our results show a strong correlation between the level of EP4 expression in Th17 cells and disease activity in patients with AS. As reported before by several other studies, we observed that the frequency of Th17 cells in the peripheral blood is elevated in patients with AS [11, 13–15]. However, we found no direct correlation between Th17 cell frequencies and disease activity as defined by the BASDAI score. A lack of correlation has been previously reported by some research groups [11, 15, 52]. In contrast, other researchers found a correlation between BASDAI scores and Th17 cell frequencies or IL-17 levels in the serum [13, 18]. The discrepancies between these reports could be explained by the fact that the amount of Th17 cells does not necessarily correlate with IL-17 concentration, since the activity of Th17 cells may differ between subjects. Moreover, IL-17 secretion is not restricted to Th17 cells and is also found in γδ T cells, natural killer cells, or lymphoid tissue inducer cells (LTi) [53]. In the facet joints of patients with spondyloarthritis, neutrophils and mast cells have been identified as additional sources of IL-17 [54]. While the level of IL-17 is therefore not useful as a surrogate marker for disease activity in AS, we suggest that the level of EP4 expression in Th17 cells could be used as a surrogate marker to discriminate between high and low disease activity. The assessment of EP4 might be helpful to obtain additional objective information on disease activity in clinical trials. Interestingly, no correlation is found between IL-17A and EP4. This can be explained by the fact that various factors have an influence on IL-17A production and on the generation of IL-17A-positive cells. Besides PGE_2_, the cytokines IL-1b, IL-6, IL-21, IL-23, and TGFβ, as well as a dietary salt intake, fatty acids, or microbiota can affect IL-17A expression [55–57]. These multiple variables might lead to a lack of correlation between IL-17A and EP4.

Th17 cell frequencies are increased in the peripheral blood of patients with AS [11, 13–15]. Our data suggest that EP4 promotes Th17 cell accumulation through upregulation of the interleukin-23 receptor (IL-23R). These findings are in line with previous observations in Th17-mediated autoimmune inflammation of the skin [25]. IL-23 is known to be implicated in the maintenance of the Th17 lineage [58]. In T cells from patients with AS, the expression levels of IL-23-regulated miRNAs are elevated [59]. In recent years, IL-23-targeting antibodies have been evaluated for the treatment of AS [60, 61]. Blocking IL-23 in combination with IL-12 has been found to be an efficient treatment for AS, while blocking IL-23 alone is inefficient [60, 61]. Therefore, additional treatment targets are required. EP4 could represent a promising target to inhibit Th17 cell development in AS. Specific EP4 antagonists have been developed and their efficiency to inhibit Th17 cells has been tested in vitro [62–64]. Selective inhibition of EP4 could represent a promising strategy to reduce Th17 cell frequencies and disease activity in AS.

## Conclusions

Our findings suggest that EP4 is implicated in the pathogenic accumulation of Th17 cells in AS and might be a potential target for new experimental treatments aiming to reduce Th17 cell activity. EP4 is significantly overexpressed in Th17 cells from patients with AS compared to healthy individuals or patients with RA. EP4-specific activation expands the Th17 cell population in vitro. Moreover, specific stimulation of EP4 induces an upregulation of IL-23R on Th17 cells, inhibits FoxO1 expression, and enhances STAT3 phosphorylation. The expression level of EP4 in ex vivo analyzed and in vitro cultured Th17 cells from patients with AS highly correlates with the BASDAI score, thereby representing a potential marker of disease activity in AS.

## Additional files

Additional file 1: **Figure S1.** Analysis of EP2 and EP4 expression in T cell subtypes. (a) Representative example of flow cytometric analysis of IL-17 and IFNγ expression in CD4^+^ T cells from patients with AS after 4 days of in vitro cell culture under Th17-skewing conditions. (b) The same cells were analyzed by RT-PCR for *IFNG* (*n* = 13 in AS and HC; *p < 0.05) and (c) *PTGER2* expression (HC *n* = 16, AS *n* = 14; n.s.). Healthy individuals served as controls (HC). (d) The percentage of EP4-positive cells was assessed by flow cytometry (HC *n* = 12; AS *n* = 14; RA *n* = 8; *p < 0.05). (e) Th17 cells from patients with RA and healthy controls were stimulated with the EP4 agonist misoprostol, prostaglandin E_2_ (PGE_2_), or the EP2 agonist butaprost for 3 days, and EP2 expression was analyzed by flow cytometry (*n* = 16; *p < 0.05). Data are shown as mean ± SEM. Mann-Whitney test was used to determine the significance. (PDF 73 kb)

Additional file 2: **Figure S2.** EP4 is not overexpressed in other CD4^+^ T cell subsets. (a) Ex vivo analysis of human CD4^+^ T cell subsets and (b) EP4 expression (HC *n* ≥ 6, AS *n* = 17, RA *n* ≥ 9, PsA *n* ≥ 6; *p = 0.05, *p* value calculated using Mann-Whitney test). (c) Cytokine expression and (d) EP4 expression in induced CD4^+^ T cell subsets after 4 days of in vitro cell culture. Th0, Th1, Th2, Th9, and iT_reg_ cells were induced from naïve CD4^+^ CD45RA^+^ CD45RO^−^ T cells (AS *n* = 8, RA *n* ≥ 4, PsA *n* = 6). (e) Relative expression of PGE_2_ receptor genes was assessed by RT-PCR (*n* = 3, *p* value calculated using Kruskal-Wallis test). Data are shown as mean ± SEM. (PDF 105 kb)

Additional file 3: **Figure S3.** EP4 is not upregulated in a positive feedback loop in other rheumatic autoimmune diseases. (a) Representative flow cytometric analysis of IL-23R expression in in vitro cultured Th17 cells from patients with AS. Th17 cells were stimulated for 3 days with the EP4 agonist misoprostol. (b) In vitro cultured Th17 cells were lysed and analyzed for their expression of EP4 by western blot. Th17 cells were stimulated for 3 days with an EP4 agonist, an EP2 agonist, or PGE_2._ One representative experiment is shown. (PDF 787 kb)

Additional file 4: **Figure S4.** Effects of PGE_2_ receptor stimulation and correlation of EP4 with disease activity. (a) RT-PCR analysis of the of *IL1B*, *IL6*, *IL21*, *IL23*, and *TGFB* genes in patients with AS after stimulation with the EP4 agonist misoprostol, PGE_2_, or the EP2 agonist butaprost for 3 days. Data are shown as relative expression (*n* = 7; *p* value calculated using Friedman test). (b) Purified CD4^+^ T cells were activated with anti-CD3 antibodies in different concentrations (1.5 μg, 5 μg, 7.5 μg) or anti-CD2/anti-CD3/anti-CD28 antibodies for 4 days or with ionomycin and PMA for 8 h and EP4 expression in IL-17^+^ CD4^+^ T cells was assessed by flow cytometry (*n* = 2). (c) Comparison of EP4 expression levels in patients with low and high BASDAI values. EP4 expression was assessed by flow cytometry in purified CD4^+^ T cells after 4 days of in vitro cell culture and is shown as percentage of positive cells (**p = 0.01, ***p = 0.001; *p* value calculated using Mann-Whitney test). (d) Correlation between BASDAI and EP4 expression in in vitro cultured Th17 cells from patients with AS (*n* = 14; *r* = 0.7591, *p* = 0.0024). (e) Correlation of BASDAI and ex vivo EP4 expression in Th17 cells from patients with AS (*n* = 13; *r* = 0.6833, *p* = 0.0122). Data are shown as mean ± SEM. Spearman *r* correlation and Mann-Whitney test were used to determine the significance. (PDF 104 kb)

## Data Availability

The datasets supporting the conclusions of this article are included within the article (and its additional files).

## Abbreviations

AS: Ankylosing spondylitis
BASDAI: Bath Ankylosing Spondylitis Disease Activity Index
CRP: C reactive protein
ESR: Erythrocyte sedimentation rate
FoxO1: Forkhead box protein O1
GWAS: Genome-wide association studies
IL-17: Interleukin-17
IL-23: Interleukin-23
IL-23R: Interleukin-23 receptor
PGE_2_: Prostaglandin E_2_
PsA: Psoriatic arthritis
RA: Rheumatoid arthritis
SLE: Systemic lupus erythematosus
Th17 cells: T helper 17 cells
TNFα: Tumor necrosis factor α

## References

[1] Evans DM, Spencer CC, Pointon JJ, Su Z, Harvey D, Kochan G (2011). Interaction between ERAP1 and HLA-B27 in ankylosing spondylitis implicates peptide handling in the mechanism for HLA-B27 in disease susceptibility. Nat Genet.

[2] Chai W, Lian Z, Chen C, Liu J, Shi LL, Wang Y (2013). JARID1A, JMY, and PTGER4 polymorphisms are related to ankylosing spondylitis in Chinese Han patients: a case-control study. PLoS One.

[3] Cortes A, Hadler J, Pointon JP, Robinson PC, Karaderi T, International Genetics of Ankylosing Spondylitis C (2013). Identification of multiple risk variants for ankylosing spondylitis through high-density genotyping of immune-related loci. Nat Genet.

[4] Tewhey R, Kotliar D, Park DS, Liu B, Winnicki S, Reilly SK (2016). Direct identification of hundreds of expression-modulating variants using a multiplexed reporter assay. Cell..

[5] Cortes A, Maksymowych WP, Wordsworth BP, Inman RD, Danoy P, Rahman P (2015). Association study of genes related to bone formation and resorption and the extent of radiographic change in ankylosing spondylitis. Ann Rheum Dis.

[6] Bowness P, Ridley A, Shaw J, Chan AT, Wong-Baeza I, Fleming M (2011). Th17 cells expressing KIR3DL2+ and responsive to HLA-B27 homodimers are increased in ankylosing spondylitis. J Immunol.

[7] van der Heijde D, Ramiro S, Landewe R, Baraliakos X, Van den Bosch F, Sepriano A (2017). 2016 update of the ASAS-EULAR management recommendations for axial spondyloarthritis. Ann Rheum Dis.

[8] van der Heijde D, Cheng-Chung Wei J, Dougados M, Mease P, Deodhar A, Maksymowych WP, et al. Ixekizumab, an interleukin-17A antagonist in the treatment of ankylosing spondylitis or radiographic axial spondyloarthritis in patients previously untreated with biological disease-modifying anti-rheumatic drugs (COAST-V): 16 week results of a phase 3 randomised, double-blind, active-controlled and placebo-controlled trial. Lancet. 2018;392(10163):2441–51. 10.1016/S0140-6736(18)31946-9 30360964

[9] Hammitzsch A, Chen L, de Wit J, Al-Mossawi MH, Ridley A, Sekine T (2018). Inhibiting ex-vivo Th17 responses in ankylosing spondylitis by targeting Janus kinases. Sci Rep.

[10] van der Heijde D, Deodhar A, Wei JC, Drescher E, Fleishaker D, Hendrikx T (2017). Tofacitinib in patients with ankylosing spondylitis: a phase II, 16-week, randomised, placebo-controlled, dose-ranging study. Ann Rheum Dis.

[11] Zhang L, Li YG, Li YH, Qi L, Liu XG, Yuan CZ (2012). Increased frequencies of Th22 cells as well as Th17 cells in the peripheral blood of patients with ankylosing spondylitis and rheumatoid arthritis. PLoS One.

[12] Mei Y, Pan F, Gao J, Ge R, Duan Z, Zeng Z (2011). Increased serum IL-17 and IL-23 in the patient with ankylosing spondylitis. Clin Rheumatol.

[13] Xueyi L, Lina C, Zhenbiao W, Qing H, Qiang L, Zhu P (2013). Levels of circulating Th17 cells and regulatory T cells in ankylosing spondylitis patients with an inadequate response to anti-TNF-alpha therapy. J Clin Immunol.

[14] Limon-Camacho L, Vargas-Rojas MI, Vazquez-Mellado J, Casasola-Vargas J, Moctezuma JF, Burgos-Vargas R (2012). In vivo peripheral blood proinflammatory T cells in patients with ankylosing spondylitis. J Rheumatol.

[15] Shen H, Goodall JC, Hill Gaston JS (2009). Frequency and phenotype of peripheral blood Th17 cells in ankylosing spondylitis and rheumatoid arthritis. Arthritis Rheum.

[16] Gracey E, Yao Y, Green B, Qaiyum Z, Baglaenko Y, Lin A (2016). Sexual dimorphism in the Th17 signature of ankylosing spondylitis. Arthritis Rheumatol.

[17] Hull DN, Cooksley H, Chokshi S, Williams RO, Abraham S, Taylor PC (2016). Increase in circulating Th17 cells during anti-TNF therapy is associated with ultrasonographic improvement of synovitis in rheumatoid arthritis. Arthritis Res Ther..

[18] Bautista-Caro MB, Arroyo-Villa I, Castillo-Gallego C, de Miguel E, Peiteado D, Puig-Kroger A (2013). Decreased Th17 and Th1 cells in the peripheral blood of patients with early non-radiographic axial spondyloarthritis: a marker of disease activity in HLA-B27(+) patients. Rheumatology (Oxford).

[19] Chen L, Al-Mossawi MH, Ridley A, Sekine T, Hammitzsch A, de Wit J (2017). miR-10b-5p is a novel Th17 regulator present in Th17 cells from ankylosing spondylitis. Ann Rheum Dis.

[20] Chen L, Ridley A, Hammitzsch A, Al-Mossawi MH, Bunting H, Georgiadis D (2016). Silencing or inhibition of endoplasmic reticulum aminopeptidase 1 (ERAP1) suppresses free heavy chain expression and Th17 responses in ankylosing spondylitis. Ann Rheum Dis.

[21] Boniface K, Bak-Jensen KS, Li Y, Blumenschein WM, McGeachy MJ, McClanahan TK (2009). Prostaglandin E2 regulates Th17 cell differentiation and function through cyclic AMP and EP2/EP4 receptor signaling. J Exp Med.

[22] Yao C, Sakata D, Esaki Y, Li Y, Matsuoka T, Kuroiwa K (2009). Prostaglandin E2-EP4 signaling promotes immune inflammation through Th1 cell differentiation and Th17 cell expansion. Nat Med.

[23] Napolitani G, Acosta-Rodriguez EV, Lanzavecchia A, Sallusto F (2009). Prostaglandin E2 enhances Th17 responses via modulation of IL-17 and IFN-gamma production by memory CD4+ T cells. Eur J Immunol.

[24] Kofler DM, Marson A, Dominguez-Villar M, Xiao S, Kuchroo VK, Hafler DA (2014). Decreased RORC-dependent silencing of prostaglandin receptor EP2 induces autoimmune Th17 cells. J Clin Invest.

[25] Lee Jinju, Aoki Tomohiro, Thumkeo Dean, Siriwach Ratklao, Yao Chengcan, Narumiya Shuh (2019). T cell–intrinsic prostaglandin E2-EP2/EP4 signaling is critical in pathogenic TH17 cell–driven inflammation. Journal of Allergy and Clinical Immunology.

[26] Maseda D, Johnson EM, Nyhoff LE, Baron B, Kojima F, Wilhelm AJ (2018). mPGES1-dependent prostaglandin E2 (PGE2) controls antigen-specific Th17 and Th1 responses by regulating T autocrine and paracrine PGE2 production. J Immunol.

[27] van der Linden S, Valkenburg HA, Cats A (1984). Evaluation of diagnostic criteria for ankylosing spondylitis. A proposal for modification of the New York criteria. Arthritis Rheum.

[28] van der Linden MP, Knevel R, Huizinga TW, van der Helm-van Mil AH (2011). Classification of rheumatoid arthritis: comparison of the 1987 American College of Rheumatology criteria and the 2010 American College of Rheumatology/European League Against Rheumatism criteria. Arthritis Rheum.

[29] World Medical A (2013). World Medical Association Declaration of Helsinki: ethical principles for medical research involving human subjects. JAMA..

[30] Schmidt A, Eriksson M, Shang MM, Weyd H, Tegner J (2016). Comparative analysis of protocols to induce human CD4+Foxp3+ regulatory T cells by combinations of IL-2, TGF-beta, retinoic acid, rapamycin and butyrate. PLoS One.

[31] Lee Y, Awasthi A, Yosef N, Quintana FJ, Xiao S, Peters A (2012). Induction and molecular signature of pathogenic TH17 cells. Nat Immunol.

[32] Laine A, Martin B, Luka M, Mir L, Auffray C, Lucas B (2015). Foxo1 is a T cell-intrinsic inhibitor of the RORgammat-Th17 program. J Immunol.

[33] Garrett S, Jenkinson T, Kennedy LG, Whitelock H, Gaisford P, Calin A (1994). A new approach to defining disease status in ankylosing spondylitis: the Bath Ankylosing Spondylitis Disease Activity Index. J Rheumatol.

[34] Calin A, Garrett S, Whitelock H, Kennedy LG, O'Hea J, Mallorie P (1994). A new approach to defining functional ability in ankylosing spondylitis: the development of the Bath Ankylosing Spondylitis Functional Index. J Rheumatol.

[35] van der Heijde D, Lie E, Kvien TK, Sieper J, Van den Bosch F, Listing J (2009). ASDAS, a highly discriminatory ASAS-endorsed disease activity score in patients with ankylosing spondylitis. Ann Rheum Dis.

[36] Lukas C, Landewe R, Sieper J, Dougados M, Davis J, Braun J (2009). Development of an ASAS-endorsed disease activity score (ASDAS) in patients with ankylosing spondylitis. Ann Rheum Dis.

[37] Reveille JD (2015). Biomarkers for diagnosis, monitoring of progression, and treatment responses in ankylosing spondylitis and axial spondyloarthritis. Clin Rheumatol.

[38] Gratacos J, Collado A, Filella X, Sanmarti R, Canete J, Llena J (1994). Serum cytokines (IL-6, TNF-alpha, IL-1 beta and IFN-gamma) in ankylosing spondylitis: a close correlation between serum IL-6 and disease activity and severity. Br J Rheumatol.

[39] Mattey DL, Packham JC, Nixon NB, Coates L, Creamer P, Hailwood S (2012). Association of cytokine and matrix metalloproteinase profiles with disease activity and function in ankylosing spondylitis. Arthritis Res Ther..

[40] Farh KK, Marson A, Zhu J, Kleinewietfeld M, Housley WJ, Beik S (2015). Genetic and epigenetic fine mapping of causal autoimmune disease variants. Nature..

[41] Zou YC, Yang XW, Yuan SG, Zhang P, Li YK (2016). Celastrol inhibits prostaglandin E2-induced proliferation and osteogenic differentiation of fibroblasts isolated from ankylosing spondylitis hip tissues in vitro. Drug Des Devel Ther.

[42] Alander CB, Raisz LG (2006). Effects of selective prostaglandins E2 receptor agonists on cultured calvarial murine osteoblastic cells. Prostaglandins Other Lipid Mediat.

[43] Xie C, Liang B, Xue M, Lin AS, Loiselle A, Schwarz EM (2009). Rescue of impaired fracture healing in COX-2−/− mice via activation of prostaglandin E2 receptor subtype 4. Am J Pathol.

[44] Fujino H, Xu W, Regan JW (2003). Prostaglandin E2 induced functional expression of early growth response factor-1 by EP4, but not EP2, prostanoid receptors via the phosphatidylinositol 3-kinase and extracellular signal-regulated kinases. J Biol Chem.

[45] Rundhaug JE, Simper MS, Surh I, Fischer SM (2011). The role of the EP receptors for prostaglandin E2 in skin and skin cancer. Cancer Metastasis Rev.

[46] Nagai S, Kurebayashi Y, Koyasu S (2013). Role of PI3K/Akt and mTOR complexes in Th17 cell differentiation. Ann N Y Acad Sci.

[47] Kurebayashi Y, Nagai S, Ikejiri A, Ohtani M, Ichiyama K, Baba Y (2012). PI3K-Akt-mTORC1-S6K1/2 axis controls Th17 differentiation by regulating Gfi1 expression and nuclear translocation of RORgamma. Cell Rep.

[48] Ma X, Aoki T, Narumiya S (2016). Prostaglandin E2-EP4 signaling persistently amplifies CD40-mediated induction of IL-23 p19 expression through canonical and non-canonical NF-kappaB pathways. Cell Mol Immunol.

[49] Margolis LM, Berryman CE, Murphy NE, Carrigan CT, Young AJ, Carbone JW (2018). PI3K-AKT-FOXO1 pathway targeted by skeletal muscle microRNA to suppress proteolytic gene expression in response to carbohydrate intake during aerobic exercise. Physiol Rep.

[50] Poloso NJ, Urquhart P, Nicolaou A, Wang J, Woodward DF (2013). PGE2 differentially regulates monocyte-derived dendritic cell cytokine responses depending on receptor usage (EP2/EP4). Mol Immunol.

[51] Adamik J, Henkel M, Ray A, Auron PE, Duerr R, Barrie A (2013). The IL17A and IL17F loci have divergent histone modifications and are differentially regulated by prostaglandin E2 in Th17 cells. Cytokine..

[52] Milanez FM, Saad CG, Viana VT, Moraes JC, Perico GV, Sampaio-Barros PD (2016). IL-23/Th17 axis is not influenced by TNF-blocking agents in ankylosing spondylitis patients. Arthritis Res Ther.

[53] Vanaudenaerde BM, Verleden SE, Vos R, De Vleeschauwer SI, Willems-Widyastuti A, Geenens R (2011). Innate and adaptive interleukin-17-producing lymphocytes in chronic inflammatory lung disorders. Am J Respir Crit Care Med.

[54] Appel H, Maier R, Wu P, Scheer R, Hempfing A, Kayser R (2011). Analysis of IL-17(+) cells in facet joints of patients with spondyloarthritis suggests that the innate immune pathway might be of greater relevance than the Th17-mediated adaptive immune response. Arthritis Res Ther..

[55] Kleinewietfeld M, Manzel A, Titze J, Kvakan H, Yosef N, Linker RA (2013). Sodium chloride drives autoimmune disease by the induction of pathogenic TH17 cells. Nature..

[56] Kim JY, Lim K, Kim KH, Kim JH, Choi JS, Shim SC (2018). N-3 polyunsaturated fatty acids restore Th17 and Treg balance in collagen antibody-induced arthritis. PLoS One.

[57] Chen L, Sun M, Wu W, Yang W, Huang X, Xiao Y, et al. Microbiota metabolite butyrate differentially regulates Th1 and Th17 cells’ differentiation and function in induction of colitis. Inflamm Bowel Dis. 2019. 10.1093/ibd/izz046. 10.1093/ibd/izz046 PMC6701512 30918945

[58] Stritesky GL, Yeh N, Kaplan MH (2008). IL-23 promotes maintenance but not commitment to the Th17 lineage. J Immunol.

[59] Lai NS, Yu HC, Tung CH, Huang KY, Huang HB, Lu MC. Aberrant expression of interleukin-23-regulated miRNAs in T cells from patients with ankylosing spondylitis. Arthritis Res Ther. 2018;20(1):259. 10.1186/s13075-018-1754-1 PMC6247500 30463609

[60] Deodhar A, Gensler LS, Sieper J, Clark M, Calderon C, Wang Y, et al. Three multicenter, randomized, double-blind, placebo-controlled studies evaluating the efficacy and safety of ustekinumab in axial spondyloarthritis. Arthritis Rheumatol. 2019;71(2):258–70. 10.1002/art.40728 30225992

[61] Baeten D, Ostergaard M, Wei JC, Sieper J, Jarvinen P, Tam LS (2018). Risankizumab, an IL-23 inhibitor, for ankylosing spondylitis: results of a randomised, double-blind, placebo-controlled, proof-of-concept, dose-finding phase 2 study. Ann Rheum Dis.

[62] Jin Y, Smith C, Hu L, Coutant DE, Whitehurst K, Phipps K (2018). LY3127760, a selective prostaglandin E4 (EP4) receptor antagonist, and celecoxib: a comparison of pharmacological profiles. Clin Transl Sci.

[63] Markovic T, Jakopin Z, Dolenc MS, Mlinaric-Rascan I (2017). Structural features of subtype-selective EP receptor modulators. Drug Discov Today.

[64] Caselli G, Bonazzi A, Lanza M, Ferrari F, Maggioni D, Ferioli C (2018). Pharmacological characterisation of CR6086, a potent prostaglandin E2 receptor 4 antagonist, as a new potential disease-modifying anti-rheumatic drug. Arthritis Res Ther..

