# Supplementary figures and images for: Prostaglandin receptor EP4 expression by Th17 cells is associated with high disease activity in ankylosing spondylitis
Source: Arthritis Res Ther. 2019 Jun 28;21:159. doi: 10.1186/s13075-019-1948-1 (PMC6599260; doi:10.1186/s13075-019-1948-1)

# Supplementary Figure S1

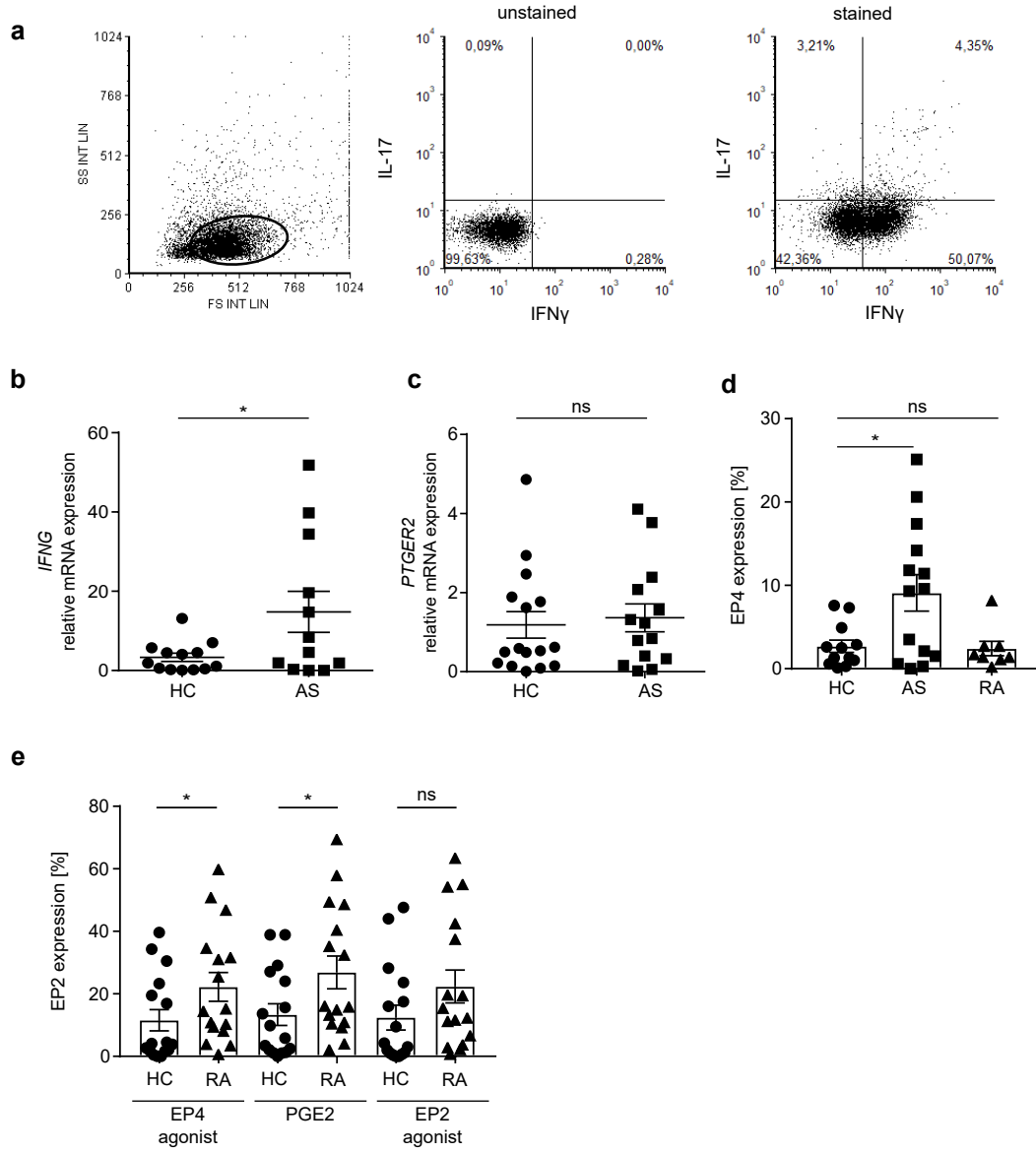

Supplement: Supplementary file 1 — Figure S1. Analysis of EP2 and EP4 expression in T cell subtypes. (a) Representative example of flow cytometric analysis of IL-17 and IFNγ expression in CD4+ T cells from patients with AS after 4 days of in vitro cell culture under Th17-skewing conditions. (b) The same cells were analyzed by RT-PCR for IFNG (n = 13 in AS and HC; *p < 0.05) and (c) PTGER2 expression (HC n = 16, AS n = 14; n.s.). Healthy individuals served as controls (HC). (d) The percentage of EP4-positive cells was assessed by flow cytometry (HC n = 12; AS n = 14; RA n = 8; *p < 0.05). (e) Th17 cells from patients with RA and healthy controls were stimulated with the EP4 agonist misoprostol, prostaglandin E2 (PGE2), or the EP2 agonist butaprost for 3 days, and EP2 expression was analyzed by flow cytometry (n = 16; *p < 0.05). Data are shown as mean ± SEM. Mann-Whitney test was used to determine the significance. (PDF 73 kb) [file 13075_2019_1948_MOESM1_ESM.pdf]

# Supplementary Figure S2

**a**

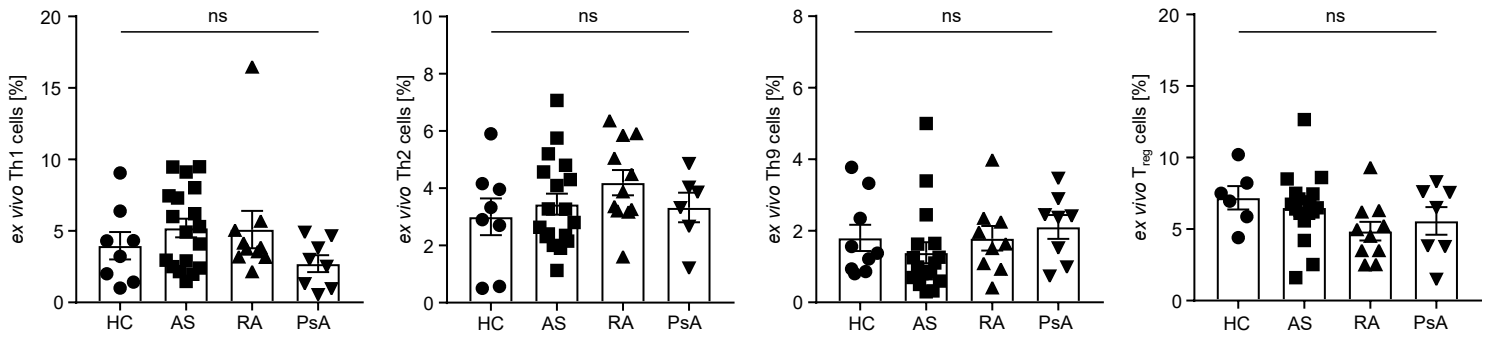

**b**

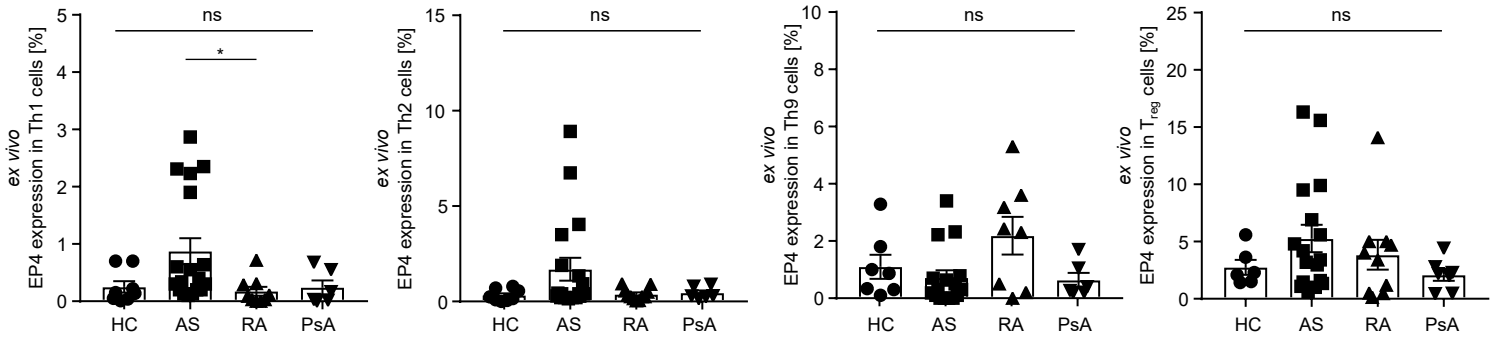

**c**

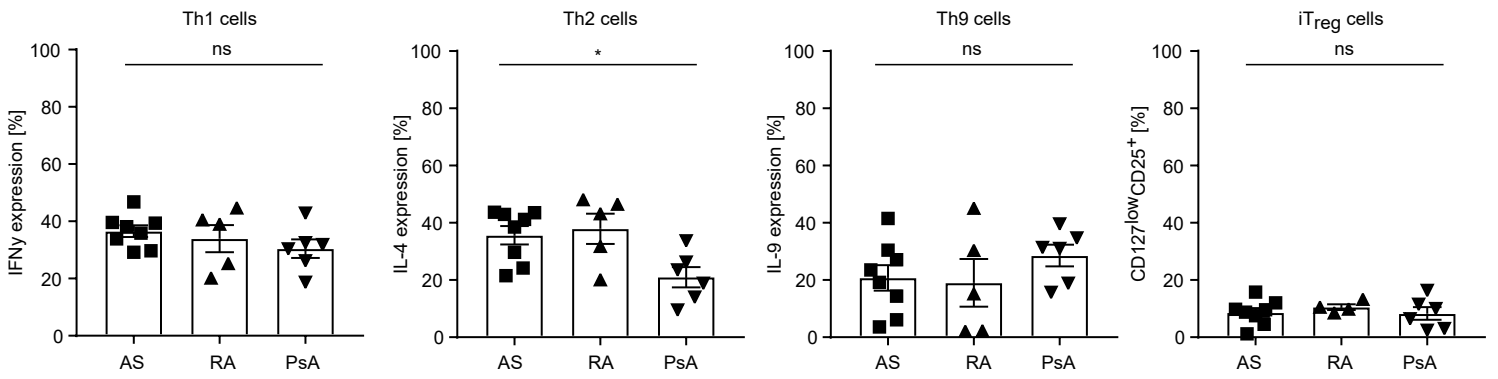

**d**

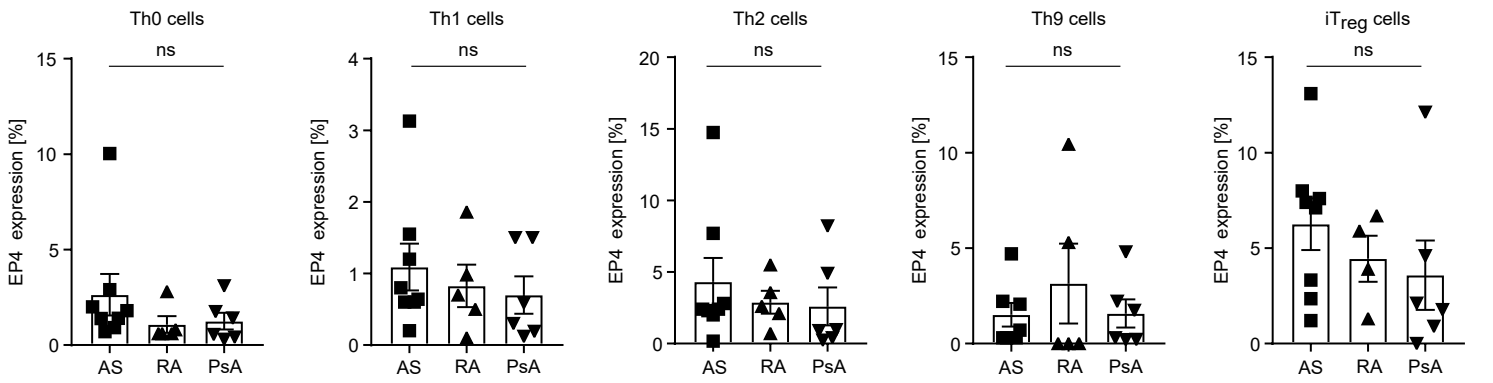

**e**

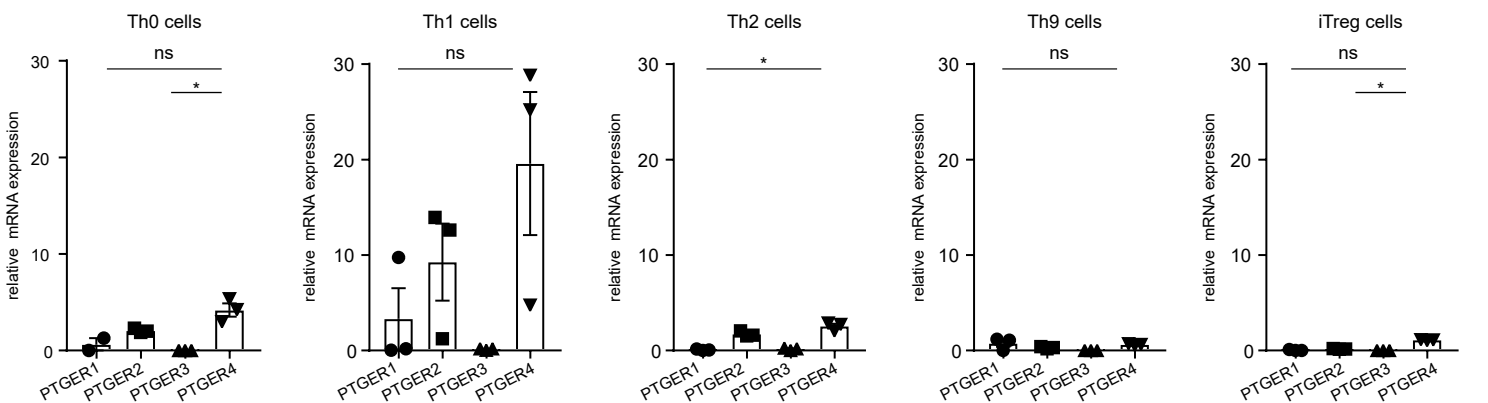

Supplement: Supplementary file 2 — Figure S2. EP4 is not overexpressed in other CD4+ T cell subsets. (a) Ex vivo analysis of human CD4+ T cell subsets and (b) EP4 expression (HC n ≥ 6, AS n = 17, RA n ≥ 9, PsA n ≥ 6; *p = 0.05, p value calculated using Mann-Whitney test). (c) Cytokine expression and (d) EP4 expression in induced CD4+ T cell subsets after 4 days of in vitro cell culture. Th0, Th1, Th2, Th9, and iTreg cells were induced from naïve CD4+ CD45RA+ CD45RO− T cells (AS n = 8, RA n ≥ 4, PsA n = 6). (e) Relative expression of PGE2 receptor genes was assessed by RT-PCR (n = 3, p value calculated using Kruskal-Wallis test). Data are shown as mean ± SEM. (PDF 105 kb) [file 13075_2019_1948_MOESM2_ESM.pdf]

Supplementary Figure S3

a

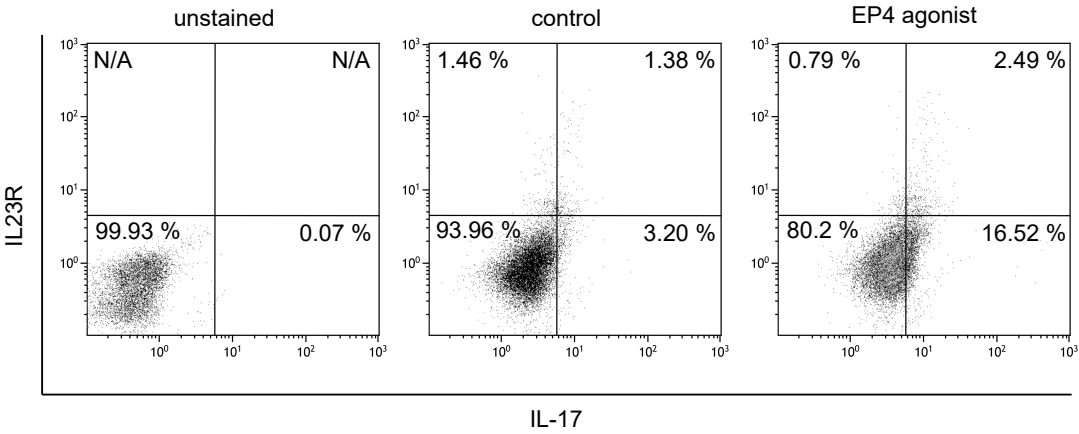

b

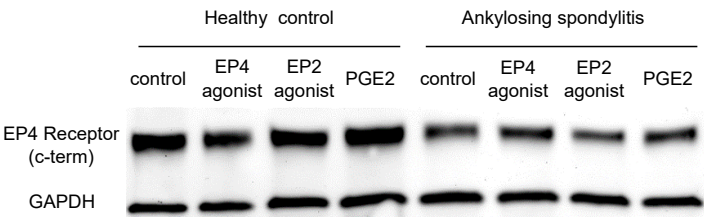

c

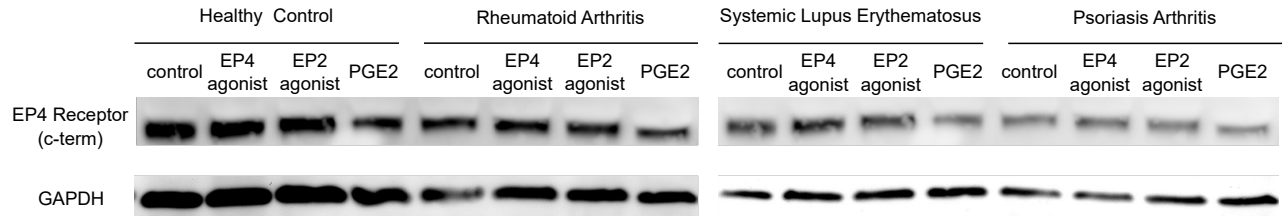

Supplement: Supplementary file 3 — Figure S3. EP4 is not upregulated in a positive feedback loop in other rheumatic autoimmune diseases. (a) Representative flow cytometric analysis of IL-23R expression in in vitro cultured Th17 cells from patients with AS. Th17 cells were stimulated for 3 days with the EP4 agonist misoprostol. (b) In vitro cultured Th17 cells were lysed and analyzed for their expression of EP4 by western blot. Th17 cells were stimulated for 3 days with an EP4 agonist, an EP2 agonist, or PGE2. One representative experiment is shown. (PDF 787 kb) [file 13075_2019_1948_MOESM3_ESM.pdf]

# Supplementary Figure S4

**a**

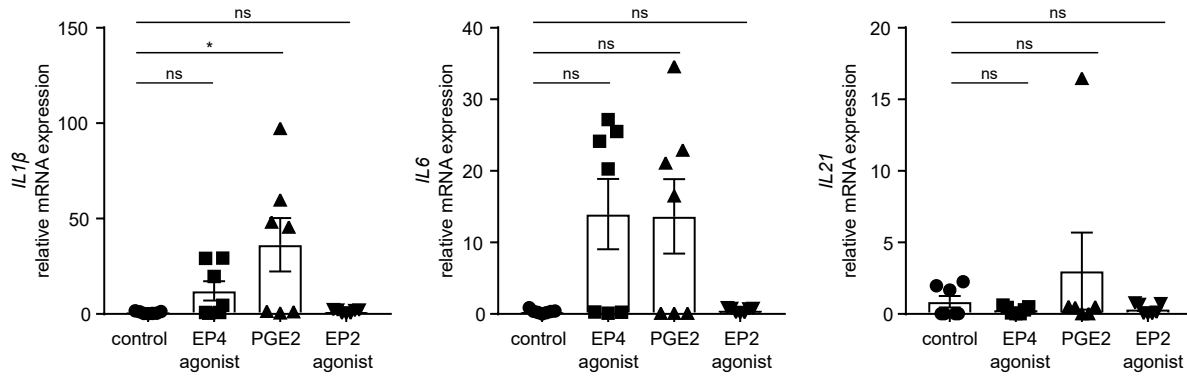

**b**

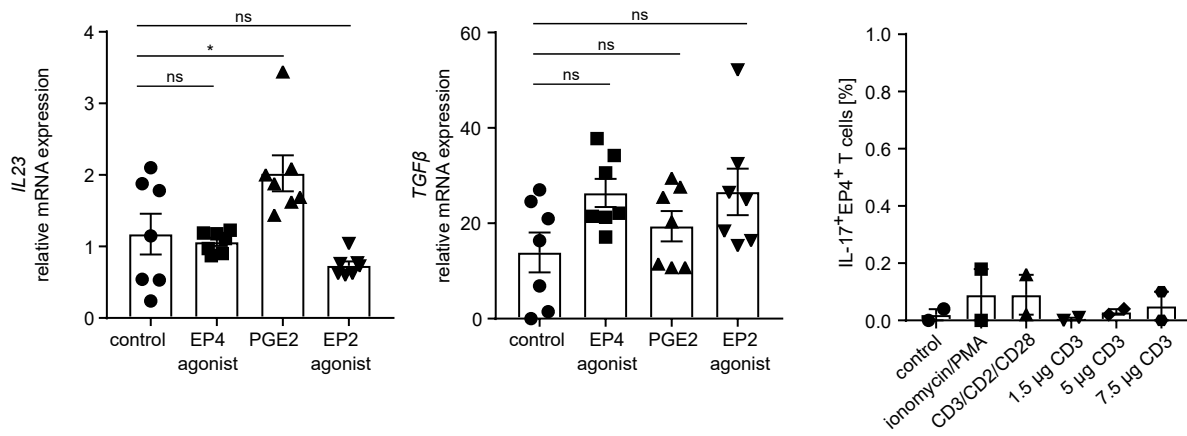

**c**

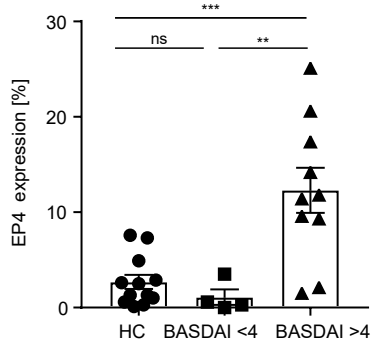

**d**

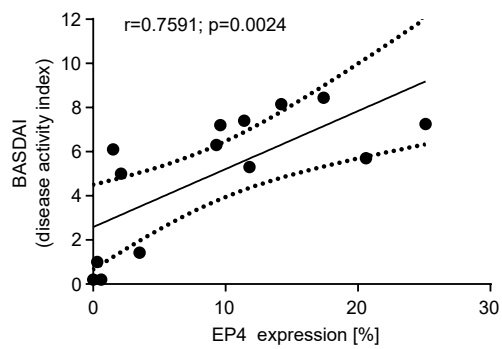

**e**

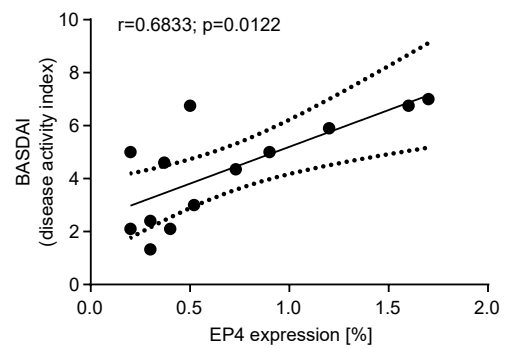

Supplement: Supplementary file 4 — Figure S4. Effects of PGE2 receptor stimulation and correlation of EP4 with disease activity. (a) RT-PCR analysis of the of IL1B, IL6, IL21, IL23, and TGFB genes in patients with AS after stimulation with the EP4 agonist misoprostol, PGE2, or the EP2 agonist butaprost for 3 days. Data are shown as relative expression (n = 7; p value calculated using Friedman test). (b) Purified CD4+ T cells were activated with anti-CD3 antibodies in different concentrations (1.5 μg, 5 μg, 7.5 μg) or anti-CD2/anti-CD3/anti-CD28 antibodies for 4 days or with ionomycin and PMA for 8 h and EP4 expression in IL-17+ CD4+ T cells was assessed by flow cytometry (n = 2). (c) Comparison of EP4 expression levels in patients with low and high BASDAI values. EP4 expression was assessed by flow cytometry in purified CD4+ T cells after 4 days of in vitro cell culture and is shown as percentage of positive cells (**p = 0.01, ***p = 0.001; p value calculated using Mann-Whitney test). (d) Correlation between BASDAI and EP4 expression in in vitro cultured Th17 cells from patients with AS (n = 14; r = 0.7591, p = 0.0024). (e) Correlation of BASDAI and ex vivo EP4 expression in Th17 cells from patients with AS (n = 13; r = 0.6833, p = 0.0122). Data are shown as mean ± SEM. Spearman r correlation and Mann-Whitney test were used to determine the significance. (PDF 104 kb) [file 13075_2019_1948_MOESM4_ESM.pdf]
